# Energy Efficiency of a Decode-and-Forward Multiple-Relay Network with Rate Adaptive LDPC Codes †

**DOI:** 10.3390/s19214793

**Published:** 2019-11-04

**Authors:** Bushra Bashir Chaoudhry, Syed Ali Hassan, Joachim Speidel, Haejoon Jung

**Affiliations:** 1 Department of Telecommunications, University of Stuttgart, 70569 Stuttgart, Germanyjoachim.speidel@inue.uni-stuttgart.de (J.S.); 2 School of Electrical Engineering & Computer Science (SEECS), National University of Sciences & Technology (NUST), Islamabad 44000, Pakistan; ali.hassan@seecs.edu.pk; 3 Department of Information & Telecommunication Engineering, Incheon National University, Incheon 22012, Korea

**Keywords:** cooperative links, decode-and-forward, energy efficiency, energy harvesting, LDPC codes, power allocation, regenerative relaying

## Abstract

This paper presents cooperative transmission (CT), where multiple relays are used to achieve array and diversity gains, as an enabling technology for Internet of Things (IoT) networks with hardware-limited devices. We investigate a channel coding aided decode-and-forward (DF) relaying network, considering a two-hop multiple-relay network, where the data transmission between the source and the destination is realized with the help of DF relays. Low density parity check (LDPC) codes are adopted as forward error correction (FEC) codes to encode and decode the data both at the source and relays. We consider both fixed and variable code rates depending upon the quality-of-service (QoS) provisioning such as spectral efficiency and maximum energy efficiency. Furthermore, an optimal power allocation scheme is studied for the cooperative system under the energy efficiency constraint. We present the simulation results of our proposed scheme, compared with conventional methods, which show that if decoupled code rates are used on both hops then a trade-off has to be maintained between system complexity, transmission delay, and bit error rate (BER).

## 1. Introduction

The Internet of Things (IoT) is a revolutionary communication paradigm, which enables the seamless integration of an excessive amount of devices (e.g., sensors, portable devices, cars, and electronic appliances) with the Internet [1]. Allowing direct interactions between devices or machines with minimal human intervention, IoT reaches various application domains such as smart homes, smart cities, healthcare, and Industry 4.0 [2,3]. However, such an interconnection between heterogenous devices is the key to paving the road for IoT as a large number of nodes in the IoT networks have very low hardware capabilities with highly limited energy. A cooperative transmission (CT) is known to be an effective solution to this issue because it provides an signal-to-noise-ratio (SNR) advantage by creating a virtual antenna array using multiple single antenna nodes [4]. This SNR advantage can be used to improve connectivity [5], throughput [6], energy balancing [7], and security [8].

The cooperative transmission with different variants gave birth to a whole new era of research in wireless communications and extensive of work has been done in this area [9,10,11,12,13,14,15,16,17,18,19,20,21,22,23,24,25,26,27,28,29,30,31,32,33,34,35], since their inception in 1971 by Van der Meulen. A simple relaying technique has already been in use in cellular networks in the form of repeaters and in satellite communications. When CT is combined with forward error correction (FEC) codes, the communication reliability can be significantly enhanced [10]. In particular, low density parity check (LDPC) codes are considered the best amongst all available FEC codes because of their capacity approaching performance and iterative decoding mechanism [11]. For this reason, there have been numerous studies on LDPC codes for CT-based networks, as in [11,12,13]. However, most existing studies on CT adopting LDPC codes have focused on the performance improvement without any consideration of link adaptation techniques. Nonetheless, some researchers have used rate adaptation techniques or more specifically, adaptive coding and modulation (ACM) in cooperative networks, but power allocation was not thoroughly considered. For example, Andreas Müller et al. in [14] and [15] discussed the decode-and-forward (DF) multihop systems with ACM, without considering any particular encoding scheme at source and/or relay(s). Moreover, the authors did not consider power allocation optimization at the transmitter. The authors in [16] adapted to different kinds of data rates and different modulation schemes in a non-FEC manner. They did not allocate a fair share of transmit power to each transmitting node. In [17], the authors considered the optimal power allocation technique under the constraint of union bound minimization. Moreover, they only considered a network with source, destination, and a single relay, where no FEC code was applied at the source or at relay. The authors in [18] considered power allocation and minimization of Chernoff bound on bit error rate (BER) performance, but did not consider FEC codes on transmitting nodes. Similarly, in [19], J. Bao et al. also considered the optimal power allocation for incremental relay selection for hybrid DF and AF relays but they also did not consider channel coding. ACM is studied in [20], where the authors study optimized power allocation for LDPC coded DF relays, however there is no code rate adaptation according to channel state information (CSI).

Hence, we delve into a unique scenario, which bridges the gap between the two groups of the researches who either considered the rate adaptation according to channel conditions or considered the optimal power allocation in practical scenarios. To be specific, we investigate a two-hop network with multiple relays where different power allocation techniques are considered under the constraint of energy efficiency. It is noted that we extend the work in the conference version of this paper [21], where a single relay was assumed, by considering a more general scenario with multiple relays, which is more realistic in IoT networks. Moreover, the system under consideration in this paper provides a comprehensive framework for optimal power allocation in a multiple relay network when variable code rates are applied according to the channel conditions. In addition, we tackle the transmit power allocation issue in such a way that it guarantees the provision of quality-of-service (QoS) in terms of lower bit error rate (BER) and maximal energy efficiency along with reduced latency. The major contributions of this work are as follows:

1. We design and analyze decoupled code rate adaptive LDPC-coded cooperative networks, where during one end-to-end transmission interval different coding rates may be used on both hops. This procedure entails the possible need for a temporary buffering of data at the involved relay stations in case the number of bits that may be forwarded with the adapted code rate on a certain hop is smaller than the number of bits received from the previous node. The achievable end-to-end performance is investigated with rigorous Monte Carlo simulations;
2. A new algorithm has been designed and developed that selects a certain code rate according to the channel conditions, with the following characteristics:
  - Only the instantaneous CSI is required for code rate adaptation;
  - The algorithm works in a distributed manner and gives individual estimate of both hops;
  - The amount of feedback overhead is kept minimal because by design the algorithm only sends a few bits as CSI to the respective source node;
3. Optimized power allocation is considered under the constraint of the network’s maximal energy efficiency and is compared with equal power allocation and with a heuristic based approach.

The remainder of this paper has been organized as follows. In Section 2, we present the system model of the wireless system under consideration along with some fundamental assumptions. In Section 3, different power allocation schemes have been discussed in certain detail. Towards the end of the paper, in Section 4, we discuss the results of different power allocation techniques through rigorous Monte Carlo simulations.

## 2. System Model

**Figure 1:**
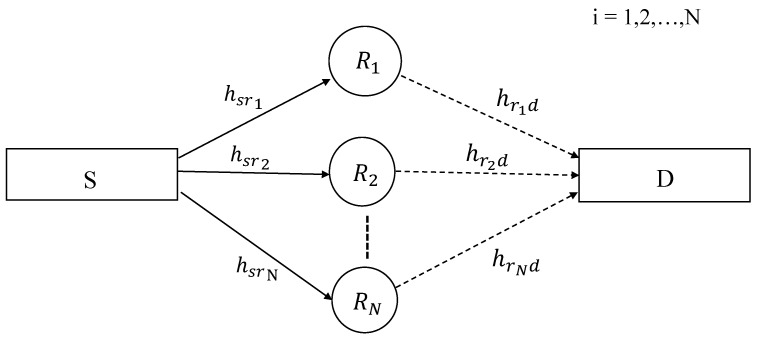
Two-hop multiple-relay network.

We consider a two-hop wireless system, with a source node *S*, a destination node *D*, and intermediate relay nodes $R_{i}$, where i∈{1,2,…,N}. These relay nodes are located at different positions between the vicinity of *S* and *D* as shown in Figure 1. There is no direct link between *S* and *D* and the source node communicates with the destination via relays only. The relays are assumed to operate in a regenerative decode-and-forward mode. A time division based half-duplex relaying has been considered, i.e., there are two phases of the data transmission. In the first phase or time slot (TS-1), *S* encodes the data with a specific code rate $C_{R}$ using LDPC codes and broadcasts it towards the relays. The relays receive the data and decode it. In the second phase or time slot (TS-2), the data is encoded at the relays with the same or varying code rates and are sent towards the destination *D*. The destination receives the data from all the relays on orthogonal channels and performs decoding. The additive white Gaussian noise (AWGN) has been assumed on all the receivers with zero mean and $N_{0}/2$ variance per real dimension.

The received signal at the $i^{th}$ relay is given as:

(1) $$y_{r_{i}}=\sqrt{P_{s}}x_{s}h_{sr_{i}}+n_{r_{i}},$$

where $x_{s}$ is the transmitted symbol from the source with the transmit power $P_{s}$. In Equation (1) the channel coefficient between the source and $i^{th}$ relay is given as $h_{sr_{i}}$ and $n_{r_{i}}$ is the additive white Gaussian noise (AWGN) sample added at the $i^{th}$ relay. It has been assumed that all the nodes are equipped with single antennas and the wireless channel on all links undergoes frequency flat fading. The channel coefficients are independent and identically distributed (i.i.d) and follow the Nakagami-*m* distribution. Moreover, we assume that all the nodes have perfect channel state information (CSI) of the links on which they receive data, hence, a perfect coherent detection is possible at the receiver.

Likewise, at the destination *D*, the received signal from the $i^{th}$ relay can be represented as:

(2) $$y_{d_{i}}=\sqrt{P_{i}}x_{r_{i}}h_{r_{i}d}+n_{d},$$

where $x_{r_{i}}$ is the transmitted symbol from the $i^{th}$ relay with transmit power $P_{i}$. $h_{r_{i}d}$ is the channel coefficient between the $i^{th}$ relay and the destination and $n_{d}$ is the AWGN sample added to the received symbol at the destination. The destination receives signals from the multiple relays on orthogonal channels.

### Data Encoding and Decoding Using LDPC Codes

We generate a (n,k) irregular LDPC code by keeping the code rate $C_{R}$ the same in an end-to-end transmission. This is the first considered scenario, which implies that same code rate is applied at the source and at all the relays irrespective of the channel conditions. $C_{R}$ is defined as $C_{R}=k/n$, where for every *k* bits of useful information, LDPC code generates a total of *n* bits, n≥k, of which (n−k) are redundant bits. In Table 1, we enlist five different code rates for an example LDPC code, which have been used in our simulation results as well.

At the source, we generate a sparse parity check matrix, H, with (n−k) rows and *n* columns. A corresponding generator matrix G is generated to encode the given sequence as $x_{s}=u\circ G$, where u is the input codeword and $x_{s}$ is the output codeword. M-ary quadrature amplitude modulation (QAM) is employed by the source in TS-1. At the relay station, *i*, the received sequence is decoded using log domain sum product algorithm (SPA). The decoded codeword $u_{i}^{\prime }$ at $i^{th}$ relay is encoded again as $x_{r_{i}}=u_{i}^{\prime }\circ G_{i}$, and after M-QAM modulation, it is transmitted by the relay in TS-2. The destination then decodes the received information using the log-domain SPA. Please note that G and $G_{i}$ may be the same or different.

After demodulation, the received vectors $y_{r_{i}}$ and $y_{d_{i}}$ constructed from Equations (1) and (2) are valid codewords only if they satisfy the parity check condition, i.e.,

(3) $${H\circ {y_{r}}_{i}}^{T}=0,$$

(4) $${H_{i}\circ {y_{d}}_{i}}^{T}=0,$$

where $H_{i}$ is the parity check matrix corresponding to $G_{i}$.

The above products in Equations (3) and (4) are zero vectors of dimension (n−k)×1. At the end, for each bit, the hard decision is made using a sum product algorithm, the SPA halts when the required number of iterations are reached or if all the parity check are satisfied.

**Figure 2:**
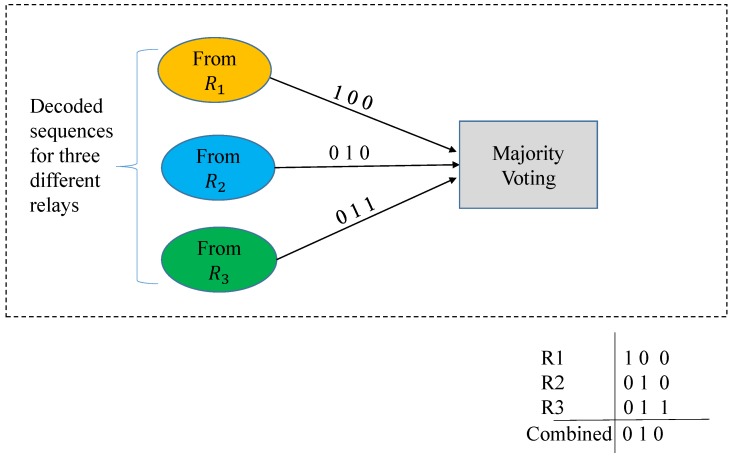
Post-detection combining with majority voting.

To improve the performance of the wireless communication links, several copies of the data coming from different paths, (here from the relays) are combined. As all the copies are combined after detection of the signals, therefore, we call it post-detection combining. In the realized scenario, the signals coming from different relays are combined at destination. The signals received from each relay are decoded separately using a LDPC decoder and are combined in a single matrix $Y_{pdc}$ of dimensions (N×k), where *N* is the number of relays. Each column of the matrix is decided on the basis of the majority voting rule, i.e., if a bit (0 or 1) has more than 50% occurrences it will be selected as the detected bit, as shown in Figure 2. It is noted that in this study, we consider an end-to-end transmission and emphasize on making an end-to-end BER performance better. Hence, even when some of the relays decode the information with higher individual BERs, when it is received at the destination, the decision is made on majority voting rule. This is also clear from Figure 2 as when the same information is received from different paths, a diversity gain is achieved. The reason for this gain is that with an increasing number of copies, the probability that all of them fade simultaneously decreases and hence, the chances of reception increase [9].

## 3. Power Allocation in Dual-Hop Networks

Power allocation (PA) is an important aspect of wireless communications and much research has been done in this regard [17,18,31,32,33,34,35]. All the transmitting terminals, be it multiple antennas, two-hop/multiple-hop relay assisted communications, or multiple-sources/multiple-relays, require different types of strategy to distribute the power to all the transmitting terminals. In multi-hop wireless networks, the instantaneous SNR, $\gamma_{i}$, at the $i^{th}$ relay can be calculated by considering the transmit power $P_{s}$, i.e.,

(5) $$\gamma_{i}=\frac{P_{s}}{\sigma^{2}}{\left|h_{sr_{i}}\right|}^{2}.$$

Energy efficiency (EE), η, is the maximum number of information bits transmitted successfully, per Joule of energy consumed at the transmitter. In other words, EE is the amount of data transmitted per unit power. Mathematically, for a single link between *S* and relay $R_{i}$, it can be defined as:

(6) $$\eta =\frac{\log_{2}\left(1+\gamma_{i}\right)}{P_{tot}}.$$

Similarly, the instantaneous SNR, $\gamma_{d_{i}}$ at *D* form a relay *i* can be defined as:

(7) $$\gamma_{d_{i}}=\frac{P_{i}}{\sigma^{2}}{\left|h_{r_{i}d}\right|}^{2},$$

where $P_{i}$ is the transmit power of relay *i* and energy efficiency at *D* can be defined as follows:

(8) $$\eta =\frac{\log_{2}\left(1+\gamma_{d_{i}}\right)}{P_{tot}}.$$

In Equations (6) and (8), $P_{tot}$ is the sum of the normalized powers at the source and all relays, i.e.,

(9) $$P_{tot}=P_{s}+\sum_{i=1}^{N}P_{i}.$$

In the following subsection we investigate the transmit power allocation under certain conditions to achieve a target BER. For discussion on energy usage and energy efficiency, a couple of different scenarios are considered where transmit power allocation is performed in different manners. The scenarios are as follows.

### 3.1. Equal Power Allocation (EPA)

Equal power allocation to all transmitting nodes reduces the computational complexity of a wireless network, keeping in view the simplicity of the power allocation algorithm. In this Section, we investigate the equal power allocation in two contexts:

1. Equal power allocation, without code rate adaptation (EPA-I).
  In this case, we assume that the same code rate is applied on both hops. Hence, the relays do not perform any temporal buffering because of the same input and output data rates. This assumption makes the implementation of such a network straightforward. For EPA-I there is no power adaptation and no code rate adaptation on the relays but equal transmit power is allocated to every transmitter regardless of its needs;
2. Equal power allocation with code rate adaptation on relays based on feedback from destination (EPA-II).
  EPA-II is also equal power allocation but code rate adaptation is done here, i.e., a higher code rate can be adapted if channel conditions are better. With bad channel conditions, a lower code rate is chosen to send the data from the relays. However, the transmit power at relays remains the same.

For EPA, in Equation (9) $P_{i}$ denotes the transmit power of the $i^{th}$ relay, under the constraint that:

(10) $$P_{i}=P_{i}^{max},\;\;\;\forall i,$$

where $P_{i}^{max}=P_{s}$, yielding $P_{i}=P_{s}$ for all *i*.

### 3.2. Optimized Power Allocation (OPA)

When equal transmit power is provided to all the transmitting nodes in network, a certain performance margin can be obtained but EPA scenarios do not maximize the system efficiency. However, this performance gap can be bridged by providing the relay nodes with an optimized power. Following that idea, we consider distributing the power in a different manner. We consider that the source *S* and all the relays $R_{i}$ share a certain pool of power, where the source always transmits with half of the total power whereas, each relay gets the fair share of transmit power on need basis. The optimized power allocation ensures the system efficiency by fair allocation of transmit power at multiple relays.

We treat this type of power allocation as an optimization problem. The transmit power is allocated in a way, such that it maximizes the energy efficiency of the system. We consider that the source always emits with a fixed transmit power $P_{s}$. The optimization problem is formulated as:

(11) $$\begin{array}{c} \begin{array}{c} \max_{P_{i}}\;\;\;\;\eta ,\;\;\;\;i=1,\ldots ,N\\ s.t\;\;\;\sum_{i=1}^{N}P_{i}\leq P_{i}^{max}, \end{array} \end{array}$$

where $P_{i}^{max}=P_{s}$.

In the above equation η is given by Equation (8). An $i^{th}$ relay can transmit with the maximum transmit power $P_{i}=P_{s}$ or a power less than $P_{s}$. However, the sum of transmit powers at all the relays should not exceed the $P_{s}$. This constraint in Equation (11) limits the maximum transmit power to maximize the EE. In case of OPA, supposedly, a relay sends with a higher code rate when the channel conditions are good. Different code rates $C_{R}$ will be selected on the basis of CSI feedback from the receiver node. We presume that a good channel condition means a high channel gain. On the basis of the waterfilling algorithm notion, we allocate more power to the channels with higher gain. Consequently, every relay gets a fair share of transmit power according to its needs. Power is distributed among multiple relays in this regard and as mentioned earlier, *S* transmits with half of the total transmit power $P_{tot}$. OPA works under the assumption that high channel gain deserves more transmit power allocation. It is different from the EPA-II methodology because there is not only the code rate adaptation on the basis of CSI feedback as in EPA-II but also that the power will be allocated on need basis. The numerical analysis of all the PA protocols we have discussed so far is presented in the next section along with simulation results.

## 4. Simulation Results and Discussion

For Monte Carlo simulations, different code lengths of LDPC codes were used but for every length *n*, 10,000 frames were delivered to the destination with 20 decoding iterations. Five different code rates were used with 4-QAM. A Nakagmi-*m* fading factor m=3 and data rate $C_{R}=1/2$ was used to collect the results, unless otherwise mentioned. We note that wherever mentioned the subscript with Nakagami-*m* factor shows whether this is the fading factor on the first or second hop.

**Figure 3:**
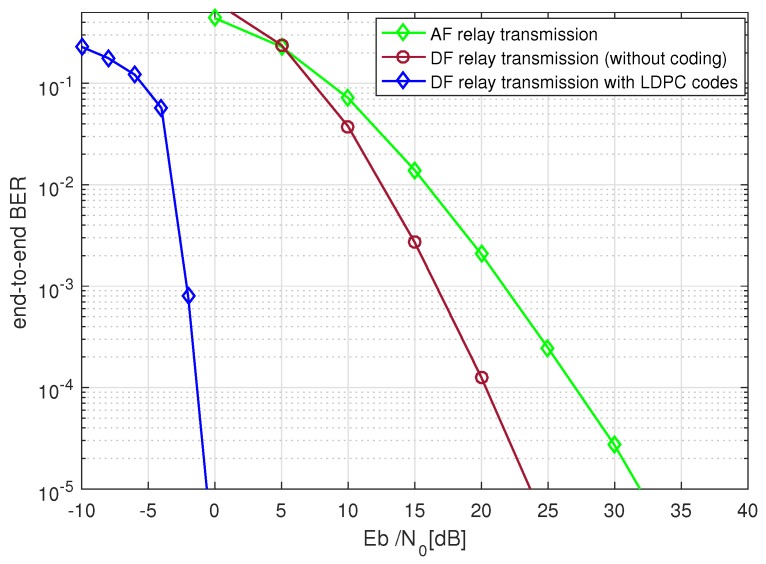
A BER (bit error rate) performance comparison between amplify-and-forward (AF), decode-and-forward (DF) coded, and non-coded relay assisted communications, LDPC code length n=648, Nakagami-*m* fading, $m_{1}=m_{2}=3$, 4-QAM (quadrature amplitude modulation), and no. of bits $=10^{5}$.

In Figure 3, a basic BER performance comparison was made where amplify-and-forward relays were studied against decode-and-forward relays. The results clearly demonstrate the supremacy of LDPC coded decode-and-forward relays. AF relaying system had the worst performance because of the well known reason of noise amplification at the relays along with the source signal. We can see that however, uncoded DF relays provided an almost 8 dB SNR gain but the LDPC coded system provided a much larger performance margin against the uncoded system. It is noted that in uncoded DF relaying the relays only perform a hard-decision decoding.

**Figure 4:**
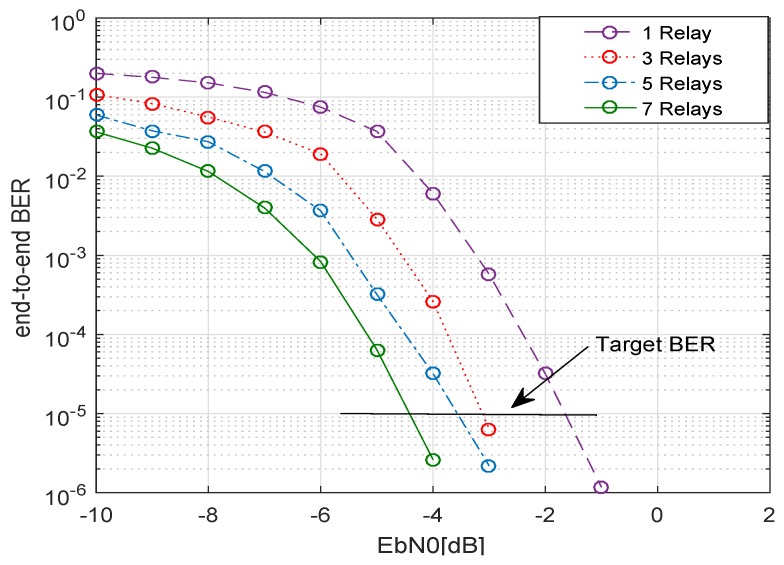
EPA-I (equal power allocation): BER of system with multiple relays with an equal transmit power, code rate 1/2, codeword length n=648, Nakagami-*m* fading, $m_{1}=m_{2}=3$, and 4-QAM.

**Figure 5:**
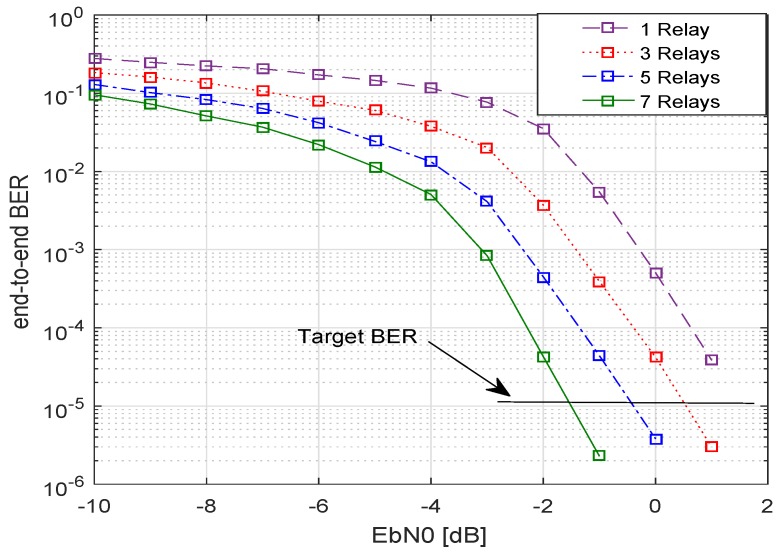
EPA-I: BER of system with multiple relays with equal transmit power (decreased by a factor of 2), code rate 1/2, codeword length n=648, Nakagami-*m* fading $m_{1}=m_{2}=3$, and 4-QAM.

In Figure 4, the BER performance for various numbers of relays is shown. As the number of relays increased, a better BER performance was achieved, e.g. for 3 relays a BER of $10^{-5}$ was gained for a SNR of −3.0 dB approximately, whereas for 7 relays the required SNR to achieve the same BER of $10^{-5}$, reduced to −4.5 dB. Figure 5 shows that if the amount of transmit power is decreased by a factor of 2 for the same system, the system performance also decreases. This can be observed by comparing Figure 4 and Figure 5.

**Figure 6:**
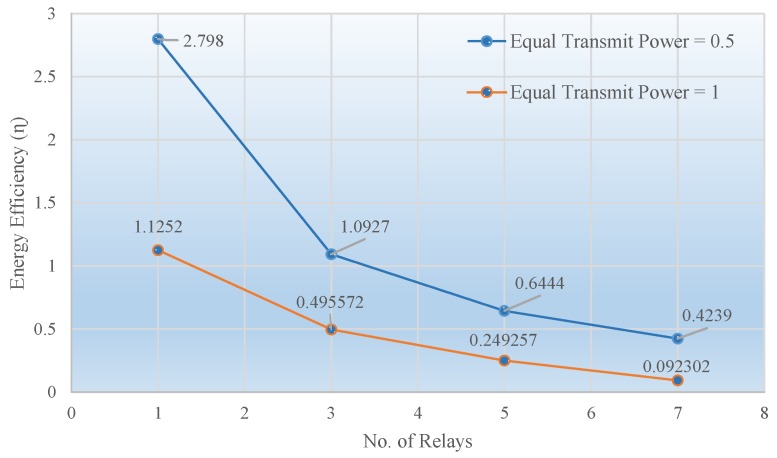
Energy efficiency vs. number of relays for target BER = $10^{-5}$, code rate 1/2, codeword length n=648, and 4-QAM.

It is evident from the two figures that when the transmit power is halved, the target BER of $10^{-5}$ for 7 relays is achieved at an SNR of about −1.5 dB. Hence, approximately a 3 dB extra SNR will be needed to achieve the target BER when transmit power is decreased by a factor of 2. Figure 6 represents the relationship between the energy efficiency of the system versus the number of cooperative relays. As the number of relays increased, the energy efficiency η dropped very quickly. This is because the total transmit power increased with the increasing number of relays, however, the data rate was not increased at the same pace. In other words, the resultant SNR to attain a certain target BER did not increase as quickly, and consequently, energy efficiency decreased. Nonetheless, this is also clear that when the transmit power was halved but assigned equally assigned, an improvement in the energy efficiency of the system could be seen.

**Figure 7:**
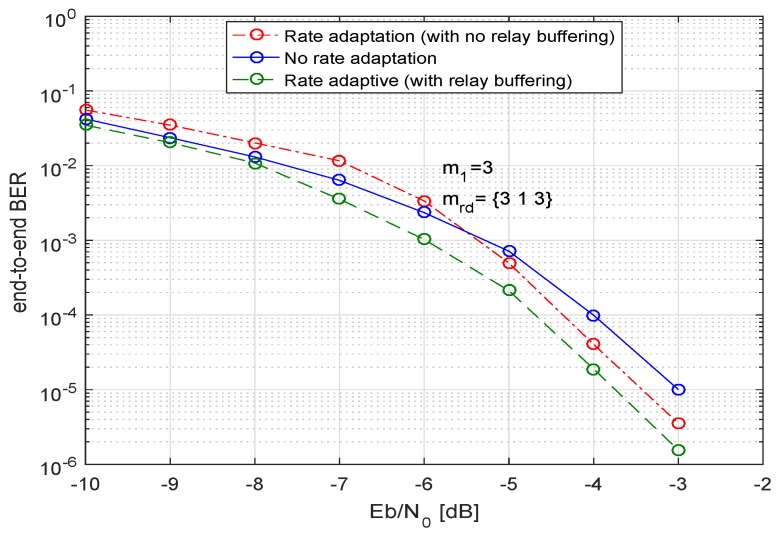
BER of multiple relays (three relays) with an equal transmit power (decreased by a factor of 2), codeword length n=648, $C_{R}$ at source 1/2, and 4-QAM.

**Figure 8:**
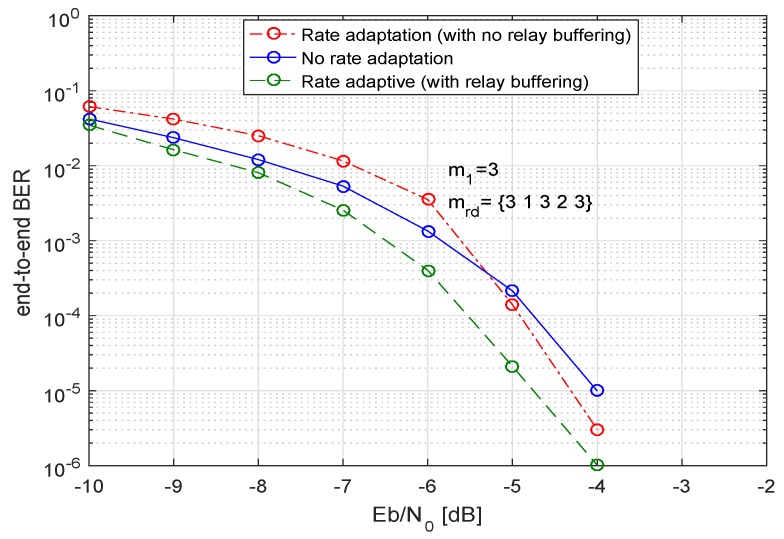
BER of multiple relays with equal transmit power = 1/2, code rate = 1/2, code length = 648, and 4-QAM.

In Figure 7 and Figure 8, we analyze our system for an optimized power allocation strategy with different conditions. In Figure 7, we can observe the three curves, the first (red) curve shows the BER performance of the system when the system adapts to the different code rates but does not do any temporal storage of the data at the relays. In this case, if a relay has to send the data at a lower code rate than the rate with which it has received the data, no bits are buffered at the relay and after sending the required bits according to the new chosen code rate, the remaining bits are disposed off.

**Figure 9:**
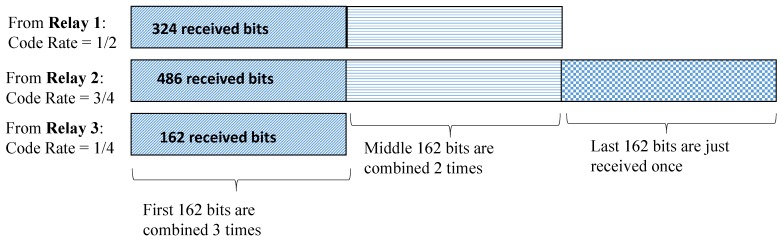
OPA (optimized power allocation) Example: Number of effective information bits, received and combined (from three relays), codeword length = 648, $m_{1}=3$, $m_{rd}=\left\{3,3,1\right\}$, source code rate 3/4, and 4-QAM.

As an example case, we consider three relays with a LDPC codeword of length n=648. We observe that if the source *S* sends data with a code rate of 3/4, then the decoded sequence length, i.e., *k* will be 486 bits. For the first relay when the relay adapts $C_{R}$ according to the channel conditions and if it adapts to a new code rate of 1/2 then it only sends 324 bits and disposes the remaining 162 information bits. Moreover, a new parity check matrix $H_{1}$ with revised parameters, *k* and n−k is defined at the relay. For the relay 2, if the same output code rate as input code rate, i.e., $C_{R}=3/4$ is chosen then no bits will be disposed off. If at relay three, only 162 information bits are transferred because of worse channel conditions on the link between relay and destination, as $m_{rd}=1$, hence, the rest of the bits are disposed off at relay three. Therefore, the destination receives the data from the first relay with a code rate of 1/2, i.e., *D* receives 324 information bits in a frame, from the second relay with a code rate of 1/4, the destination receives 162 bits and from the third relay with a code rate of 3/4, and *D* receives a frame of length 486 bits. Consequently, when all three frames have received from the relays, they are of different lengths. In Figure 9 we have outlined the whole scenario. We observe that by disposing the remaining bits, the system can avoid the overhead of buffering the information at relay(s). Furthermore, there are lower delays in the information transmission. Moreover, the destination combines the only bits that are received in the same time-slot on orthogonal channels from all the relays. Please note, we have used the Nakagami-*m* factor as $m_{1}$ for the first hop, i.e., for the channels between source and multiple relays and it is denoted as $m_{rd}$ for the links between relays and destination. Moreover, the above example addresses a rather generic scenario and does not necessarily use the same parameters as for the simulation results in Figure 7.

Furthermore, in Figure 7, the second (blue) curve demonstrates the BER performance of the system when there is no code rate adaptation, the input code rate and output code rate at the relay are the same. The last (green) curve shows the bit-error-rate performance when the system applies the code rate adaptation based on channel conditions and also buffers the remaining bits at the relay. In this case, not only are the bits temporarily stored at the relay but they are also sent using the extra time-slots. As a result the destination receives the complete sequence from each relay in multiple time-slots. Then *D* applies the post-detection combining on all the sequences received from relays. If we compare all three curves, we see that at the start the worst performance is shown by the system which applies the code rate adaptation without buffering any information at the relays. Still, at a higher SNR this curve surpasses the performance of the code rate non-adaptive system and shows a very close performance to the system that does a temporal buffering of data at the relays, the reason being that because of the multiple relays, the destination receives multiple copies of the data sequences. If some of the information sequences are of not of the same length, the system can still achieve a good diversity gain. This fact is more evident when the number of relays increases. The advantages of this approach are manifold, i.e.,

1. No temporal buffering of the data at the relays;
2. Lesser processing at the relays;
3. Lesser power consumption, as less information has to be sent;
4. Shorter time delays.

Hence, the OPA approach where the code rate adaptation is done with no intermediate data buffering at relays but post detection combining is done on sequences of unequal lengths, appears to be a promising solution for multiple relays. Moreover, this approach also ensures the maximal energy efficiency by allocating the optimal share of transmit power to the transmitting nodes.

**Figure 10:**
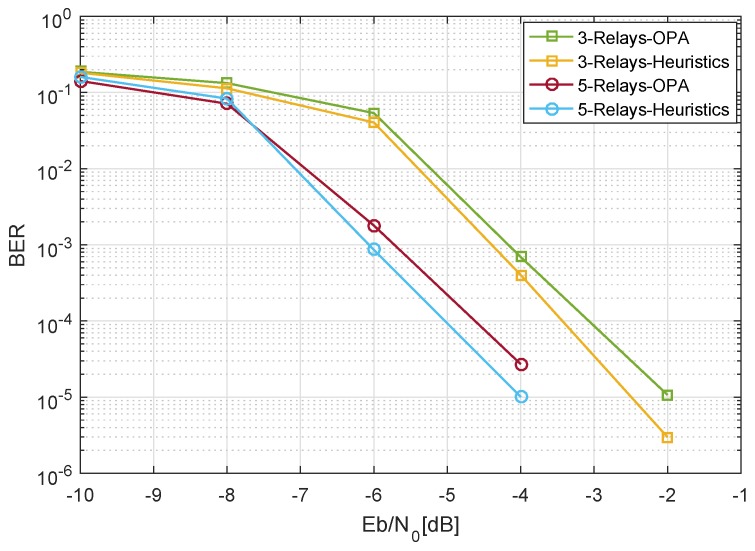
OPA compared with heuristics based approach, LDPC codeword length = 312, $m_{1}=m_{2}=3$, 4-QAM, and $C_{R}=1/2$.

In Figure 10, we compare the optimized power allocation technique with a heuristics based approach. The heuristics based method provides the proof of concept that the OPA strategy studied in this research works in the close proximity of the heuristic approach. It is less complex as compared to the heuristics based approach. Nonetheless, the proposed OPA provides a good solution that is energy efficient due to the same power budget of the system in all the cases.

**Figure 11:**
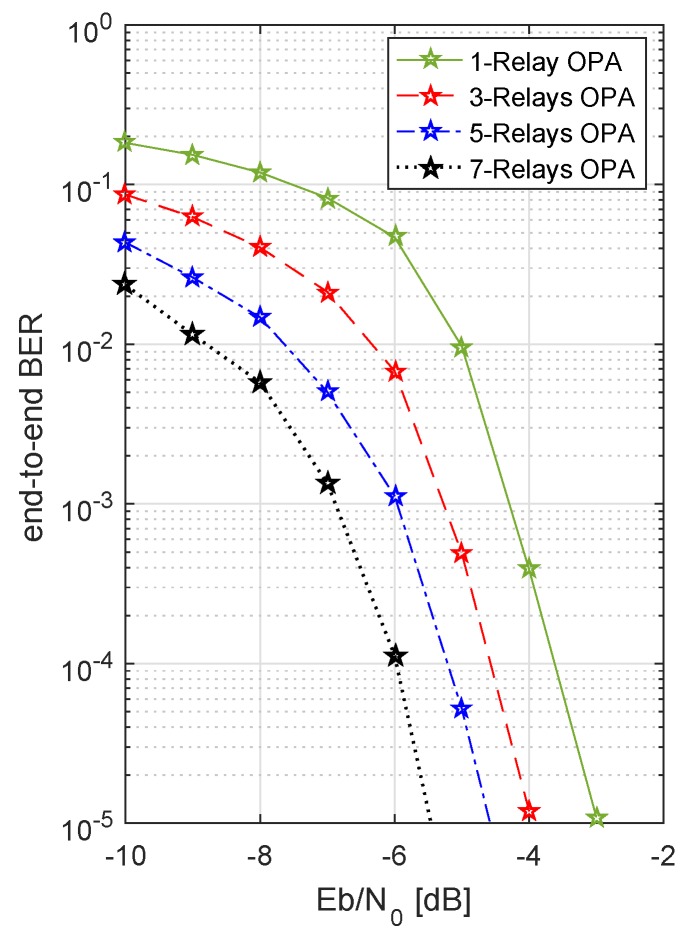
OPA: BER of system with multiple relays, codeword length = 312, $m_{1}=m_{2}=3$, 4-QAM, and varying code rates $C_{R}$.

**Figure 12:**
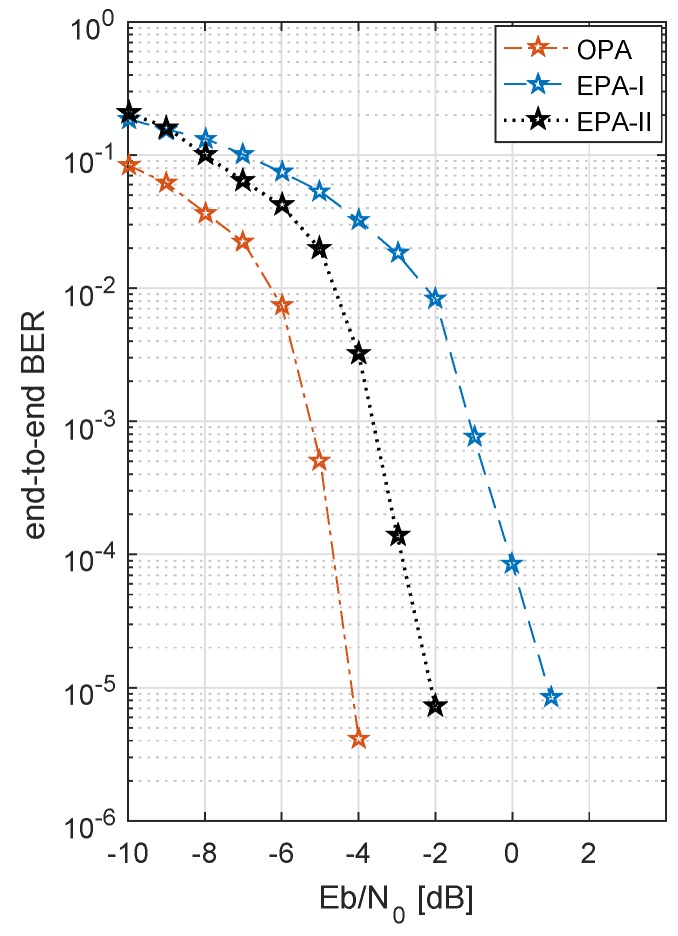
BER of multiple relays (five relays) with three PA (power allocation) schemes, (independent but not identically distributed (n.i.i.d) channels), and 4-QAM.

Figure 11 shows the results of optimized power allocation and adaptive code rates. An identical Nakagami-*m* factor is considered on both hops, on all channels between source and multiple relays and relays and destination, i.e., $m_{1}=m_{2}=3$. This is clear from Figure 11 that when transmit power is allocated optimally, with an increasing number of relays, the BER performance becomes better and better, because of the diversity gain. As the number of paths providing the independent information increases, a diversity gain is achieved, which is actually the change in the slope of BER curve. Hence, the probability of errors decreases with the increasing number of relays. This is worth mentioning here that in Figure 11 the diversity gain curves look more like the power gain curves, but in fact the total power budget for all the cases was kept the same and the additional gain is achieved because of the additional diversity. Figure 12 shows the performance of three different PA schemes studied in this work. As shown in Figure 12, results are collected for n.i.i.d Nakagami fading, i.e., value of fading factor *m* between two hops is different. OPA provides a performance gain of ∼5 dB and ∼2 dB as compared to EPA-I and EPA-II, respectively. From the discussion, EPA-I and EPA-II are simpler strategies of power allocation, as compared to the OPA. As, like in EPA-I, there is no transmit power adaptation in EPA-II and only the code rates adaptation is done under the CSI feedback. Table 2 presents an overview of the PA strategies. Positively, OPA not only improves the BER performance of the system but also maximizes the energy efficiency of the system. As we can see, OPA is the superior power allocation technique because it takes into account the real time channel conditions, calculates the instantaneous SNR, and maximizes the energy efficiency of the system.

Furthermore, when there is a code rate adaptation for the relay to destination link, a considerable gain can be attained with post-detection combining even without buffering the information at the intermediate relays. This method not only has many advantages over the technique with information buffering at the relays, but it also reduces the complexity of the overall system and helps the relays to consume less power, reducing the overall system delay.

## 5. Conclusions

In this paper a DF-based two-hop multiple-relay network was studied as a solution to enhance IoT network performances with the limited node capabilities. Complex and computation intensive operations were carried out at relays. The scenario under consideration could increase the hardware and software complexity of the relays because relays have to implement the complex algorithms of code rate and transmit power adaptation. However, relays with some extra computing and storage capabilities could reduce the load of the base stations and central control nodes. In this work, we considered the transmit power allocation in different manners, at source and at relays. It was observed that optimized power allocation was the best method to enhance the BER performance under the constraint of energy efficiency.When there was code rate adaptation for the relay to destination link, a considerable gain could be attained without buffering the information at the intermediate relays. This method not only has many advantages over the technique with information buffering at the relays, but it also reduces the complexity of the overall system, helping the relays to consume less power and reduces the overall system delay. Future extensions of this work could include more sophisticated power allocation algorithm to further improve the performance with more practical constraints such as limited CSI feedback and energy balancing.

## Figures and Tables

**Table 1:** Different data rates $C_{R}$ for LDPC (low density parity check), code length n= 312.

Code Rate	Info. Bits	Check Bits	CodeRate Index
$C_{R}$	k	(n−k)	$r_{\mathrm{index}}$
1/4	78	234	0
1/2	156	156	1
3/4	234	78	2
5/6	260	52	3
7/8	273	39	4

**Table 2:** Overview of three power allocation strategies.

	EPA-I	EPA-II	OPA
Code rate adaptation	×	✓	✓
Power adaptation	×	×	✓

## Abbreviations

The following abbreviations are used in this manuscript:

IoT	Internet of Things
CT	Cooperative transmission
SNR	Signal-to-noise-ratio
FEC	Forward error correcting
LDPC	Low density parity check
DF	Decode-and-forward
QoS	Quality-of-service
EE	Energy efficiency
BER	Bit error rate
CC	Cooperative communications
SNR	Signal-to-noise ratio
ACM	Adaptive coding and modulation
TS	Time slot
AP	Access point
AWGN	Additive white Gaussian noise
i.i.d	Independent and identically distributed
CSI	Channel state information
QAM	Quadrature amplitude modulation
SPA	Sum product algorithm
PA	Power allocation
EPA	Equal power allocation
OPA	Optimized power allocation
